# CA125 test result, test-to-diagnosis interval, and stage in ovarian cancer at diagnosis: a retrospective cohort study using electronic health records

**DOI:** 10.3399/BJGP.2020.0859

**Published:** 2021-04-20

**Authors:** Garth Funston, Luke TA Mounce, Sarah Price, Brian Rous, Emma J Crosbie, Willie Hamilton, Fiona M Walter

**Affiliations:** The Primary Care Unit, Department of Public Health and Primary Care, University of Cambridge, Cambridge.; University of Exeter Medical School, University of Exeter, Exeter.; University of Exeter Medical School, University of Exeter, Exeter.; National Cancer Registration and Analysis Service, Public Health England, Cambridge.; Gynaecological Oncology Research Group, University of Manchester; consultant in gynaecological oncology, Manchester University NHS Foundation Trust, Manchester.; University of Exeter Medical School, University of Exeter, Exeter.; The Primary Care Unit, Department of Public Health and Primary Care, University of Cambridge, Cambridge.

**Keywords:** CA125, cancer antigen 125, diagnostic intervals, early diagnosis, general practice, ovarian cancer

## Abstract

**Background:**

In the UK, the cancer antigen 125 (CA125) test is recommended as a first-line investigation in women with symptoms of possible ovarian cancer.

**Aim:**

To compare time between initial primary care CA125 test and diagnosis, tumour morphology, and stage in women with normal (<35 U/ml) and abnormal (≥35 U/ml) CA125 levels prior to ovarian cancer diagnosis.

**Design and setting:**

Retrospective cohort study using English primary care and cancer registry data.

**Method:**

Associations between CA125 test results and test-to-diagnosis interval, stage, and ovarian cancer morphology were examined.

**Results:**

In total, 456 women were diagnosed with ovarian cancer in the 12 months after having a CA125 test. Of these, 351 (77%) had an abnormal, and 105 (23%) had a normal, CA125 test result. The median test-to-diagnosis interval was 35 days (interquartile range [IQR] 21–53) for those with abnormal CA125 levels, and 64 days (IQR 42–127) for normal CA125 levels. Tumour morphology differed by CA125 result: indolent borderline tumours were less common in those with abnormal CA125 levels (*n* = 47, 13%) than those with normal CA125 levels (*n* = 51, 49%) (*P*<0.001). Staging data were available for 304 women with abnormal, and 77 with normal, CA125 levels. Of those with abnormal CA125 levels, 35% (*n* = 106) were diagnosed at an early stage, compared to 86% (*n* = 66) of women with normal levels. The odds of being diagnosed with early-stage disease were higher in women with normal as opposed to abnormal CA125 levels (odds ratio 12.2, 95% confidence interval = 5.8 to 25.1, *P*<0.001).

**Conclusion:**

Despite longer intervals between testing and diagnosis, women with normal, compared with abnormal, CA125 levels more frequently had indolent tumours and were more commonly diagnosed at an early stage in the course of the disease. Although testing approaches that have greater sensitivity might expedite diagnosis for some women, it is not known if this would translate to earlier-stage diagnosis.

## INTRODUCTION

Ovarian cancer is the sixth most common cancer to affect women in the UK, with >7000 women diagnosed each year.^1^ It has the worst prognosis of all gynaecological cancers, accounting for >4000 UK deaths annually.^2^ Although, overall, ovarian cancer prognosis is relatively poor, this varies markedly based on tumour type: studies conducted in the US and Sweden report that 5-year relative survival rates are 48% for invasive epithelial cancer (the most common type), compared with 93% for ovarian germ-cell tumours and 97% for borderline tumours.^3^^,^^4^

Most women with ovarian cancer are diagnosed after presenting with symptoms in primary care. However, the symptoms — such as bloating and abdominal pain — are non-specific and, therefore, have relatively low positive predictive values for the disease.^5^^,^^6^ In 2011, the National Institute for Health and Care Excellence (NICE) advocated testing for the serum biomarker cancer antigen 125 (CA125) in women with symptoms of possible ovarian cancer in primary care.^7^ NICE recommended that women with an elevated CA125 (≥35 U/ml) should undergo ultrasound testing;^7^ however, they did not provide guidance on the follow-up or investigation of women with ‘normal’ (<35 U/ml) CA125 levels. Many other countries — including Ireland, Australia, Canada, and the US — also recommend CA125 as a primary care test for ovarian cancer.^8^

CA125 is a glycoprotein found in healthy ovaries, but blood levels commonly increase in ovarian cancer; around 80% of women with ovarian cancer have raised CA125 levels pre-surgery.^9^ CA125 is more frequently elevated in advanced, rather than early-stage, disease and in some tumour types than others.^10^ Concerns have been expressed that using CA125 as a single first-line investigation might delay diagnosis and lead to worse outcomes in women whose ovarian cancer is not associated with CA125 levels ≥35 U/ml,^11^ yet there is little research exploring the relationship between CA125, time to diagnosis, and outcomes.

In this study, the authors examined the association of initial primary care prediagnostic CA125 results with the time between testing and diagnosis (test-todiagnosis interval), tumour morphology, and disease stage in women with ovarian cancer.

## METHOD

### Study design, setting, and data sources

This retrospective cohort study utilised data from the Clinical Practice Research Datalink (CPRD) GOLD database, a dataset containing postcode-linked deprivation measures (provided by CPRD), and data from the National Cancer Registration and Analysis Service (NCRAS), which acts as the English cancer registry. CPRD GOLD comprises anonymised, coded primary care data, including laboratory results and diagnoses, for around 7% of the UK population.^12^ The deprivation dataset consists of a five-level Townsend score — an area-level deprivation metric, in which higher scores indicate greater material deprivation. NCRAS data consists of detailed information on cancers diagnosed in England, including stage and morphology.^13^ CPRD–NCRAS linkage was performed at patient level by NHS Digital.^14^

**Table:** How this fits in

Cancer antigen 125 (CA125) is used as an initial test for women who present to primary care with symptoms of possible ovarian cancer, but research has shown that CA125 levels are normal in 23% of women prior to diagnosis. In the present study it was found that, although women with normal CA125 test results take longer to receive a diagnosis after testing than those with abnormal results, they are more likely to have less aggressive, more-curable forms of disease, and be diagnosised at an earlier cancer stage. This provides some reassurance for those using, and being tested for, CA125. However, improving the sensitivity of primary care testing approaches for ovarian cancer could still be of benefit to patients.

In order to match the coverage of NCRAS, this study was restricted to England.

### Study period and cohort

A data sample obtained for a related study^15^ was used. The sample consisted of women with a CA125 test recorded in CPRD GOLD between 1 May 2011 and 31 December 2014. From this sample, the following were excluded:

- women aged <18 years;
- those registered at a GP practice not deemed by the CPRD to be ‘up to standard’ regarding data quality;^12^
- those with a record of ovarian cancer on, or before, the CA125 test date; and
- women who had a CA125 test in the 12 months prior to the first CA125 test during the study period.

In order to maximise data quality, only CA125 entries recorded in standard CA125 units (U/ml, IU/ml, KU/L, or KIU/L) and with a laboratory upper reference limit were accepted. Similarly, CA125 values associated with clearly erroneous upper reference limits (such as 245 U/ml, 420 U/ml, and 455 U/ml) were excluded, as these could also indicate issues with the recording or coding of CA125 values.^15^ The authors then identified women who had been diagnosed with ovarian cancer, as recorded in NCRAS data, within 12 months of CA125 testing. This group formed the study cohort.

Ovarian cancer, on the basis of codes from the tenth revision of the International Classification of Diseases (ICD-10), was defined as an ovarian malignancy (C56), a fallopian tube malignancy (C57.0), a peritoneal malignancy (C48.1 and C48.2), or a neoplasm of uncertain behaviour of the ovary (D39.1).^15^ Fallopian and peritoneal cancers arise from the same tissue type and are diagnosed, staged, and treated in the same way as cancer arising from the surface of the ovary.

Borderline tumours are non-invasive, usually diagnosed at an early stage, and have a good prognosis. However, these may recur and, generally, require surgery. Borderline tumours are included in NICE guidance on ovarian cancer detection.^7^

### CA125 category

NICE recommends using a CA125 cut-off of 35 U/ml.^7^ Therefore, women were classified on the basis of the initial CA125 test into two groups:

- abnormal: CA125 level of ≥35 U/ml; and
- normal: CA125 level of <35 U/ml.

### Covariates

A code list was used to identify symptoms of possible ovarian cancer included in current NICE guidelines^16^ — namely, abdominal/pelvic pain, abdominal distension/bloating, change in bowel habit, fatigue, weight loss, urinary frequency/urgency, loss of appetite, pelvic mass, or ascites — that had been recorded in CPRD GOLD in the 30 days prior to CA125 testing. Level of deprivation was determined using the five-level Townsend score in the deprivation measures dataset.

### Test-to-diagnosis interval

The date of cancer diagnosis is recorded for all tumours in NCRAS data. The test-todiagnosis interval (days from first CA125 test in the year before diagnosis to diagnosis date, as recorded in NCRAS data) was calculated for all women.

### Cancer stage and morphology

Tumour behaviour, morphology, and stage were identified from the NCRAS data. Tumours were classified on the basis of ICD-10 codes as: ‘borderline epithelial’, ‘invasive epithelial’, ‘invasive non-epithelial’, and ‘invasive not otherwise specified (NOS)’. Stage was categorised as early (stage I–II) or late (stage III–IV).

### Statistical analysis

Accelerated failure time (AFT) models were used to examine the association between CA125 test results and test-to-diagnosis intervals. AFT models are a parametric time-to-event analysis previously utilised in CPRD research.^17^ AFT models can be used to calculate time ratios. A time ratio >1 indicates that a variable prolongs the time to an event (for example, diagnosis), whereas a ratio <1 indicates that the variable reduces the time to the event. A univariate model was constructed to examine the relationship between the CA125 test result and test-todiagnosis interval. A multivariable model was constructed incorporating age, a binary variable denoting the presence/absence of relevant symptoms prior to CA125 testing, and Townsend score. The presence or absence of a symptom was included as there is evidence that symptoms are more likely to be coded, rather than recorded in free text (which is unavailable for research), when they are more severe/persistent — which could result in expedited referral and diagnosis.^18^ Weibull, generalised gamma, log-normal, and log-logistic distributions were examined. Log-logistic distribution was the best-fit parameterisation, according to the Akaike information criterion. Time ratios with associated *P*-values and 95% confidence intervals (CIs) were reported.

Fisher’s exact test was used to assess whether women with abnormal and normal CA125 test results differed significantly in tumour morphology. Pairwise analyses were then performed to assess whether there was a statistically significant difference for each morphology category. The authors corrected for multiple comparisons, setting the significance level at *P* = 0.01.^19^

In a subgroup for whom stage data were recorded, logistic regression was used to examine the association between the CA125 test result and the disease stage at diagnosis. Adjustments were made for age, the presence/absence of a recorded symptom, and the Townsend score. Given the favourable prognosis of borderline tumours, a subanalysis was performed that excluded these. The authors explored the relationship between explanatory variables with missing stage data using logistic regression. Crude and adjusted odds ratios (ORs) with 95% CIs and associated *P*-values are reported.

All analyses were performed using Stata (version 15.1).

## RESULTS

**Figure 1.**
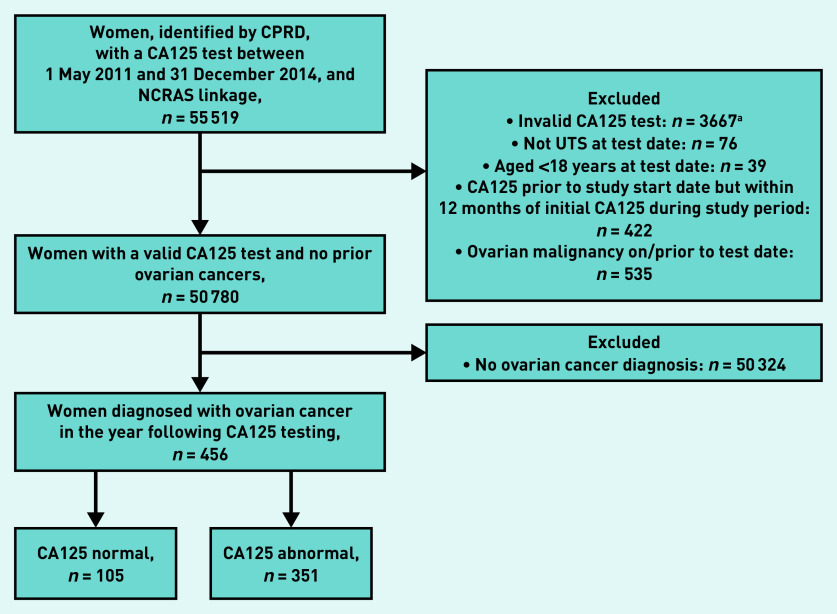
***Application of selection criteria.*** *^a^****No CA125 value, no or incorrect units, or no or spurious upper threshold recorded. CA125 = cancer antigen 125. CPRD = Clinical Practice Research Datalink. NCRAS = National Cancer Registration and Analysis Service. UTS = up to standard.***

The CPRD provided data on 55 519 women who were eligible for NCRAS linkage and who had a CA125 test between 1 May 2011 and 31 December 2014. After exclusions, 456 women diagnosed with ovarian cancer in the 12 months following CA125 testing were included in the study (Figure 1). Of these, 105 women (23%) had a normal initial CA125 result and 351 (77%) an abnormal CA125 result. A total of 41 (9%) women had a repeat CA125 test performed prior to diagnosis. Thirty women with an abnormal initial CA125 test result had a repeat test; for 29 (97%) of these, the result of the repeat test was also abnormal. Eleven women with a normal initial CA125 test had a repeat test and eight (73%) of these had an increase in their CA125 level; however, in only three cases (27%) was this increase sufficient to reach the ≥35 U/ml threshold (data not shown).

Mean age was higher in those with abnormal CA125 test results than those with normal CA125 test results, and a greater proportion of women with abnormal CA125 test results had a coded symptom of possible ovarian cancer (Table 1).

**Table 1.** Patient groups and baseline characteristics

**CA125 test result**	***n***	**Mean age at diagnosis, years (range)**	**Patients with a symptom of possible ovarian cancer recorded pre-testing, *n* (%)^a^**	**Townsend score, *n* (%)**				
				**Level 1**	**Level 2**	**Level 3**	**Level 4**	**Level 5**
Abnormal	351	65 (22–93)	212 (60)	80 (23)	100 (28)	78 (22)	61 (17)	32 (9)
Normal	105	57 (18–87)	59 (56)	24 (23)	31 (30)	25 (24)	14 (13)	11 (10)
Overall cohort	456	63 (18–93)	271 (59)	104 (23)	131 (29)	103 (23)	75 (16)	43 (9)

*CA125 = cancer antigen 125.*

### Test-to-diagnosis interval

The overall median test-to-diagnosis interval in the cohort was 42 days (interquartile range [IQR] 25–62) (data not shown). The interval was 35 days (IQR 21–53) for those with abnormal CA125 test results and 64 days (IQR 42–127) for those with normal CA125 test results (Table 2). AFT models demonstrated a statistically significant association between CA125 test results and the test-to-diagnosis interval. A time ratio of 2.0 (95% CI = 1.7 to 2.4, *P*<0.001) indicated that the test-to-diagnosis interval for those women with normal CA125 test results was twice as long as for those with abnormal CA125 test results. The time ratio remained unaltered when adjusting for age, the presence/absence of a recorded symptom, and Townsend score.

**Table 2.** Median intervals by CA125 test result, and crude and adjusted associations between CA125 test result and test-to-diagnosis interval

**CA125 test result**	***n***	**Median test-to-diagnosis interval in days, *n* (IQR)**	**Unadjusted association**		**Adjusted association^a^**	
			**Time ratio (95% CI)**	***P*-value**	**Time ratio (95% CI)**	***P*-value**
Abnormal	351	35 (21–53)	Reference	<0.001	Reference	<0.001
Normal	105	64 (42–127)	2.0 (1.7 to 2.4)	—	2.0 (1.6 to 2.4)	—

a *Adjusted for age, presence/absence of a recorded symptom, and Townsend score. Individual associations for all variables are displayed in Supplementary Table S1. CA125 = cancer antigen 125. IQR = interquartile range.*

### Tumour morphology

Tumour morphology differed significantly, in statistical terms, by CA125 result (*P*<0.001) (Table 3). Invasive epithelial cancers were the most common type in women with abnormal CA125 test results (81%), whereas borderline tumours were the most common type in women with normal CA125 test results (49%). Serous tumours accounted for 52% of invasive tumours in those with an abnormal CA125 test result, compared with 30% in those with a normal CA125 test result (data not shown).

**Table 3.** Tumour morphology by CA125 test result

	***n***	**Borderline tumour, *n* (%)**	**Invasive tumour**			**Overall analysis, *P*-value**
			**Epithelial, *n* (%)**	**Non-epithelial, *n* (%)**	**NOS, *n* (%)**	
**Abnormal CA125 test result**	351	47 (13)	284^a^ (81)	4 (1)	16 (5)	<0.001
**Normal CA125 test result**	105	51 (49)	39^b^ (37)	9 (9)	6 (6)	—
**Pairwise analysis, *P*-value**	—	<0.001	<0.001	<0.001	0.6	—

a *Serous,* n *= 158; endometrioid,* n *= 16; mucinous,* n *= 14; clear cell,* n *= 14; other epithelial,* n *= 13; epithelial cancers of unknown morphology,* n *= 69.*

b *Serous,* n *= 16; endometrioid,* n *= 4; mucinous,* n *= 8; clear cell,* n *= 3, other epithelial,* n *= 4; epithelial cancers of unknown morphology,* n *= 4. P-values are derived from Fisher’s exact test for independence. CA125 = cancer antigen 125. NOS = not otherwise specified, that is, could not be classified as epithelial or non-epithelial based on the information in the cancer registry.*

### Stage at diagnosis

Staging information was missing for 75 women: 47 with an abnormal CA125 test result and 28 with a normal CA125 test result. In women with an abnormal CA125 test result in whom stage was recorded (*n* = 304), 106 (35%) were diagnosed with early-stage disease. In women with a normal CA125 test result in whom stage was recorded (*n* = 77), 66 (86%) were diagnosed with early-stage disease (data not shown).

Logistic regression, performed on data for patients with recorded disease stage and adjusted for age, the presence/absence of a recorded symptom, and Townsend score demonstrated that the odds of being diagnosed with early-stage disease were 12.2 times higher in women with normal than abnormal CA125 test results (Table 4). A subanalysis conducted after excluding borderline tumours demonstrated a statistically significant association between having a normal CA125 test result and being diagnosed at an early stage (OR 9.0, 95% CI = 4.0 to 19.8) (see Supplementary Table S2).

**Table 4.** The association between CA125 test results, age, and the presence/absence of a recorded symptom with early (stage I–II) diagnosis

**Variable**	***n***	**Unadjusted**		**Adjusted^a^**	
		**OR (95% CI)**	***P*-value**	**OR (95% CI)**	***P*-value**
Abnormal CA125 test result	304	Reference	—	Reference	—
Normal CA125 test result	77	11.2 (5.7 to 22.1)	<0.001	12.2 (5.8 to 25.5)	<0.001
Age	—	0.95 (0.93 to 0.96)	<0.001	0.94 (0.92 to 0.96)	<0.001
No symptom record	148	Reference	—	—	—
Symptom record	233	0.51 (0.33 to 0.77)	0.001	0.35 (0.21 to 0.59)	<0.001

a *Model also adjusted for Townsend score. Data not shown for Townsend score as the variable was statistically insignificant (*P *= 0.9). CA125 = cancer antigen 125. OR = odds ratio.*

There was strong evidence to support an association between having a normal CA125 test result and having missing cancer stage at diagnosis in a logistic regression model; no such association was identified when borderline tumours were excluded from analysis (data not shown).

## DISCUSSION

### Summary

Women with normal CA125 test results in primary care, prior to receiving a diagnosis of ovarian cancer, took twice as long to be diagnosed following testing as those with abnormal CA125 test results. Despite this, in women for whom staging data were available, 86% of those with normal CA125 test results were diagnosed at an early stage compared with only 35% of those with abnormal CA125 test results. In addition, indolent borderline ovarian tumours were more common, and aggressive invasive epithelial cancers were less common, in women with normal CA125 test results than in women with abnormal CA125 test results.

### Strengths and limitations

A major strength of this study is its large size — the sample is equivalent to >6% of all ovarian cancers diagnosed in the UK each year. The results should be generalisable to women tested for CA125 in primary care prior to ovarian cancer diagnosis, as the primary care database used is generally representative of the UK population.^12^ In addition, ovarian cancer diagnoses were identified from NCRAS, which reports a near-100% case ascertainment.^13^

This study does, however, have some limitations. When defining the cohort, it was assumed that cancer diagnosed within 12 months of the initial CA125 test was present at the time of testing. A period of 1 year, which has been used in similar studies^15^^,^^20^^,^^21^ and was specified prior to data analysis,^15^ was chosen as a compromise between minimising the inclusion of incidental cancers and maximising the inclusion of relevant cancers. Examining a longer follow-up was not possible as NCRAS data were only available until the end of 2015. However, given that only one woman out of 456 was diagnosed in month 12, extending follow-up is unlikely to alter the results. A shorter follow-up period — for example, 6 months — was not examined as this would have preferentially excluded patients from the group with normal CA125 test results (who have longer test-diagnosis intervals); the results would, therefore, have been biased.

Patients with severe disease, who often have severe symptoms, frequently experience expedited diagnoses when compared with those with less severe disease — an observation sometimes referred to as the ‘sick quick’ phenomenon.^22^ As CA125 levels are also more likely to be elevated in women with more-severe disease, this may act as a confounder. The analyses were adjusted for the presence/absence of relevant coded symptoms, as symptoms may be more likely to be coded (rather than mentioned in free text) if they are more severe,^18^ but it is unlikely that it was possible to adjust fully for severity of symptoms and disease.

The authors considered adjusting for ethnicity in the analyses, but this was not done as not all patients have an ethnicity recorded in CPRD GOLD.^23^ The authors are not aware of any evidence within the literature indicating that ethnicity is associated with either diagnostic interval or stage at diagnosis for ovarian cancer, and would not expect the inclusion of ethnicity to markedly alter the results.

There was a statistically significant association between having a normal CA125 result and having missing stage at diagnosis. This is to be expected, as stage is less frequently recorded in the cancer registry for borderline tumours, which are more common in women with normal CA125 test results. It is reassuring that when borderline tumours were excluded no statistically significant association between the CA125 result and missing stage was identified, and a normal CA125 result was still strongly associated with early-stage diagnosis. Although there is no reason to suspect that study findings would differ markedly if staging data were available for all patients, the magnitude of the association between CA125 result and stage should be interpreted with caution.

### Comparison with existing literature

Previous research has identified an association between false negative results and longer healthcare intervals: in one study, patients with a negative chest X-ray who went on to be diagnosed with lung cancer experienced longer primary care intervals than those with an abnormal chest X-ray;^24^ in another study, patients with a false negative rheumatoid factor in primary care, prior to a rheumatoid arthritis diagnosis, took longer to be referred to a specialist.^25^ Research indicates that receiving an ‘all clear’ diagnosis (no cancer) following testing in primary care can provide reassurance to patients, which can lead to delayed re-presentation if symptoms persist or recur.^26^ Similarly, false reassurance could affect GPs, prompting them to seek alternative diagnoses and delaying referral.^24^^,^^27^

Few studies have investigated the relationship between false negative results and cancer outcomes in patients who are symptomatic, although one — by Yeh *et al* ^28^ — did find that patients with false negative fine-needle aspiration results, who were then diagnosed with thyroid cancer, were more likely to have vascular and capsular invasion and experience persistent disease post-treatment.

In the study presented here, for the majority of women with normal CA125 test results, cancer was detected at an early stage; this was in contrast with women with abnormal results, despite those with normal CA125 test results having longer test-todiagnosis intervals. This finding could be due to differences in tumour type. In the study presented here, borderline tumours were nearly four times as common in women with normal, rather than abnormal, levels of CA125. Borderline tumours less frequently cause elevations in CA125 than their invasive counterparts, tend to grow slowly, and 80% are diagnosed at an early stage.^29^ In contrast, invasive epithelial tumours, which typically have an insidious onset and poor survival, were twice as common in women with abnormal than normal CA125 levels. Further, aggressive invasive serous tumours, which are more frequently diagnosed at a later stage and more frequently elevate CA125 levels than other invasive tumour types,^10^ accounted for half of invasive tumours in women with abnormal CA125 test results and only a third of invasive cancers in women with normal CA125 test results.

The authors employed the NICE advocated threshold of 35 U/ml in the present study to categorise results as ‘normal’ or ‘abnormal’. However, this is an oversimplification: recent research has shown that the probability of ovarian cancer is much higher in women with a CA125 level of 34 U/ml compared with those with a CA125 level of 1 U/ml,^15^ yet these results are all classified as ‘normal’ under NICE guidelines (and within this study). Newly developed CA125-based primary care prediction models could help select women for further investigation or referral (instead of the 35 U/ml threshold),^15^ but require further evaluation.

### Implications for research and practice

CA125 test results detected 77% of ovarian cancer cases in the cohort and 88% of the invasive epithelial subtype, which is responsible for the majority of ovarian cancer mortality.^30^ Abnormal CA125 test results are, therefore, helpful in identifying women with possible ovarian cancer, especially the most lethal type. However, a normal CA125 test result does not exclude disease.

It is reassuring that most women with normal CA125 test results were diagnosed at an early stage, despite taking longer to be diagnosed. However, given the observational nature of this study, it was not possible to determine to what extent women with normal CA125 test results experienced disease progression or worse survival rates as a result of their prolonged test-to-diagnosis intervals. Diagnostic strategies that use novel serum biomarkers or imaging modalities in combination with CA125 may detect additional ovarian cancer cases,^8^^,^^31^ which could expedite diagnosis in some women. However, large, prospective studies would need to be undertaken to determine whether implementing more sensitive testing strategies would lead to earlier stage diagnosis and improved survival.

Regardless of its impact on survival, reducing unnecessary delay in ovarian cancer diagnosis is likely to be beneficial for women with normal CA125 test results. Delay in cancer diagnosis is associated with psychological distress, particularly among women,^32^ and perceived delays can damage doctor–patient relationships.^33^ Earlier diagnosis of ovarian cancer could reduce morbidity, even if a stage shift is not achieved, by detecting lower-volume disease.^31^ Possible strategies to reduce diagnostic delay could include appropriate safety netting with reassessment, and undertaking re-testing or alternative investigations (for example, ultrasound), if symptoms persist or worsen. In the study presented here, only a small proportion of women with normal results in their initial CA125 test had a repeat test. In 73% of repeat tests, there was an increase in CA125 levels, but in only 27% was this increase sufficient to reach the 35 U/ml threshold — this supports the idea that rising levels below the 35 U/ml threshold could be used to prompt further investigation.^31^ The nature, duration, and severity of presentation should also be considered when deciding on a follow-up strategy. For example, if a patient develops a pelvic mass (which has a high positive predictive value for ovarian cancer) an urgent referral is warranted,^16^^,^^34^ whereas alternative follow-up strategies, such as referral for ultrasound or CA125 re-testing, may be more appropriate for less highly predictive presentations.
